# Unmet healthcare needs of elderly people in Korea

**DOI:** 10.1186/s12877-018-0786-3

**Published:** 2018-04-20

**Authors:** Yoon-Sook Kim, Jongmin Lee, Yeonsil Moon, Kyoung Jin Kim, Kunsei Lee, Jaekyung Choi, Seol-Heui Han

**Affiliations:** 1 Department of Quality Improvement, Konkuk University Medical Center, 120-1 Neungdong-ro (Hwayang-dong), Gwangjin-gu, Seoul, 05030 South Korea; 2 Department of Rehabilitation Medicine, Konkuk University School of Medicine, Konkuk University Medical Center, 120-1 Neungdongro (Hwayang-dong), Gwangjin-gu, Chungju, 05030 South Korea; 3 Department of Neurology, Konkuk University School of Medicine, Konkuk University Medical Center, 120-1 Neungdong-ro (Hwayang-dong), Gwangjin-gu, Seoul, 05030 South Korea; 4 Department of Family Medicine, Konkuk University School of Medicine, Konkuk University Medical Center, 120-1 Neungdong-ro (Hwayang-dong), Gwangjin-gu, Seoul, 05030 South Korea; 5 Department of Preventive Medicine, Konkuk University School of Medicine, 268 Chungwon-daero Chungju-si Chungcheongbuk-do, Chungju, 27478 South Korea

**Keywords:** Unmet healthcare needs, Elderly, Aging

## Abstract

**Background:**

Elderly people often have more complicated healthcare needs than younger adults due to additional functional decline, physical illness, and psychosocial needs. Unmet healthcare needs increase illness severity, complications, and mortality. Despite this, research on the unmet healthcare needs of elderly people is limited in Korea. This study analysed the effect of functional deterioration related to aging on unmet healthcare needs based on the Korea Health Panel Study.

**Methods:**

This cross-sectional study used data from the 2011–2013 survey of 8666 baseline participants aged 65 years and older. Unmet healthcare needs were calculated using a complex weighted sample design. Group differences in categorical variables were analysed using the Rao-Scott Chi-square test. Using logistic regression analysis, the association between unmet healthcare needs and aging factors was analysed.

**Results:**

The prevalence of unmet healthcare needs in Korean elderly was 17.4%. Among them, the leading reason was economic hardship (9.2%). Adjusting for sex, age, socioeconomic characteristics, and health-related characteristics, the group with depression syndrome was 1.45 times more likely to have unmet healthcare needs than that without depression syndrome (95% CI = 1.13–1.88). The group with visual impairment was 1.48 times more likely to have unmet healthcare needs than that without it (95% CI = 1.22–1.79). The group with hearing impairment was 1.40 times more likely to have unmet healthcare needs than that without it (95% CI = 1.15–1.72). The group with memory impairment was 1.74 times more likely to have unmet healthcare needs than that without it (95% CI = 1.28–2.36).

**Conclusions:**

The unmet medical needs of the elderly are more diverse than those of younger adults. This is because not only socioeconomic and health-related factors but also aging factors that are important to the health of the elderly are included. All factors were linked organically; therefore, integrated care is needed to improve healthcare among the elderly. To resolve these unmet healthcare needs, it is necessary to reorganize the healthcare system in Korea to include preventive and rehabilitative services that address chronic diseases in an aged society and promote life-long health promotion.

## Background

‘Unmet healthcare needs’ is defined as the lack of services judged necessary to avoid negative health consequences [1, 2]. Reasons for unmet healthcare needs are classified into three categories: availability of services (e.g. waiting time before receiving care and services not available in a required area), accessibility (cost, transportation, etc.), and acceptability of available services (attitudes toward and knowledge about health care, etc.) [3, 4]. As of 2013, approximately 3% of the population of Europe have unmet healthcare needs owing to cost, waiting time before receiving care, and services not available in the required area [5].

In Korea, the medical insurance system was extended to all citizens in 1989, which improved access to medical services. However, a high share of out-of-pocket expenses due to the limited range and level of benefits covered served as a barrier to access to medical services [6]. The ability to pay for medical expenses, such as sharing of out-of-pocket expenses, is an important factor in meeting medical needs, especially among those of lower socioeconomic status [3, 7]. Many studies have suggested an association between socioeconomic status and healthcare use [8–11]. Everyone wants to live happily and be healthy, and the key to maintaining good health is to use medical services without delay when medical service is needed [12].

South Korea has the fastest growing aged population in the world [13]. It is expected to become an ‘aging society’ in 2017 and a ‘post-aged society’ in 2026 [13]. South Korean women are projected to have a 90% probability of living over 86 years in 2030, which is the same as the highest worldwide life expectancy in 2012, and a 57% probability of living over 90 years [14].

However, these elderly people require more medical services. Problematically, retirement and decreased income are likely to lead to unmet medical care. Elderly people often have more complicated needs compared with younger adults because of additional functional decline, physical illness, and psychosocial needs [15]. Unmet healthcare needs also increase illness severity, complications, and mortality [2, 16]. Despite these alarming results, research on the unmet healthcare needs of elderly people has been limited in Korea.

Older adults have diminished physical functions and mental abilities, which reduce their ability to adapt to the environment. Moreover, the physical degradation of the brain decreases elderly adults’ cognitive functioning, which not only impairs their ability to perform activities of daily living but also leads to social isolation [17]. This deterioration of functioning is associated with unmet healthcare needs, which, in turn, aggravates older adults’ health further. Consequently, we analysed the effect of this functional deterioration on older adults’ unmet healthcare needs based on data from the Korea Health Panel Study (KHPS).

## Methods

### Study population

The KHPS is conducted annually by the Korea Institute for Health and Social Affairs and the National Health Insurance Service. The KHPS is conducted to produce basic data on the utilization of health care, medical expenditures, health status, and behaviour in Korea. The KHPS surveys nationally representative households in South Korea by computer-assisted personal interviewing. Sampling was done using a two-stage, stratified, cluster extraction method with probability proportionality. The KHPS started in 2008, and unmet healthcare needs have been measured continuously since 2011; therefore, we used data from the 2011–2013 KHPS. Our sample was restricted to individuals aged 65 years or older.

### Measures

The KHPS provides a variety of information on medical use behaviour and medical expenditures. The independent variables used in this study were variables that were related to medical use in previous studies [3, 15, 18]. Our researchers thought that aging factors were critical factors in the unmet healthcare needs among the elderly. Therefore, we classified the aging factors separately into health-related factors. The aging factors were composed mainly of physiological changes of aging, as presented by Chang et al. [19].

The variables used in this study were sex, age, socioeconomic factors (spouse, education, types of health insurance, private health insurance, economic status, and household income), health-related factors (self-perceived health status, current smoker, chronic disease [according to the Korean Standard Disease/Sickness Classification, KCD-6], usual source of care, and regular family doctor), and aging factors (disability, depressive symptoms, visual impairment, hearing impairment, memory impairment, and decision-making impairment). The variables are described in Table 1.

**Table 1:** Variables description

Variables	Description
Unmet healthcare needs	During the past 12 months, was there a time when you did not receive the medical service you needed? ‘*yes*’ or ‘*no*’
Reasons for unmet healthcare needs	If the response about unmet healthcare needs is ‘*yes*’, why? (1) economic hardship, (2) inaccessible transportation, (3) physical disabilities, (4) problems finding childcare, (5) mild symptoms, (6) lack of information about hospitals, (7) lack of available time, (8) difficulties in getting appointments at hospitals, (9) no regular family doctor, and (10) other. According to previous research [21], the reasons for unmet healthcare needs were classified into three categories: ‘economic hardship’ (economic hardship), ‘scheduling conflict’ (lack of available time) and ‘other reasons’ (inaccessible transportation, physical disabilities, problems finding childcare, mild symptoms, lack of information about hospitals, difficulties in getting appointments at hospitals, no regular family doctor, and other).
Household income	Household income calculated and equalized by taking the square root of the number of household members. Household income was divided into five quintiles, the 1st quintile is the lowest quintile (lowest 20%), and the 5th quintile is the highest (highest 20%) quintile. In this study, household income was classified into three categories: ‘1st quintile’, ‘2nd quintile’, and ‘3rd quintile or more’.
Current smoker	Do you currently smoke? Responses were rated on a 4-point scale: (1) *currently smoking every day,* (2) *sometimes smoking,* (3) *former,* and (4) *never.* In this study, current smoker was classified into two categories: ‘yes’ (currently smoking every day and sometimes smoking) and ‘no’ (former and never).
Usual source of care	When you are sick or need advice from a doctor for your health, are there any medical institutions that you usually visit? ‘*yes*’ or ‘*no*’
Regular family doctor	When you are sick or need advice about your health, do you have a doctor who visits often? ‘*yes*’ or ‘*no*’
Disability	Have you ever been diagnosed with a disability from a doctor? ‘*yes*’ or ‘*no*’
Depressive symptoms	Did you have difficulty in daily life due to feeling sad or unhappy for more than two weeks in the past year? ‘*yes*’ or ‘*no*’
Visual impairment	Do you have a vision difficulty? Responses were rated on a 4-point scale: 1 (*never*) to 4 (*always*). In this study, visual impairment was classified two categories: ‘yes’ (2–4) and ‘no’ (1).
Hearing impairment	Do you have a hearing difficulty? Responses were rated on a 4-point scale: 1 (*never*) to 4 (*always*). In this study, visual impairment was classified two categories: ‘yes’ (2–4) and ‘no’ (1).
Memory impairment	Do you have difficulty in daily life due to mental confusion or memory loss? ‘*yes*’ or ‘*no*’
Decision-making impairment	Are you having difficulty making decisions that hinder your daily life? ‘*yes*’ or ‘*no*’

### Analysis

The advantage of the panel survey is that as the sample size increases, the degree of freedom increases, which improves the efficiency of the estimator and reduces the collinearity problem between the explanatory variables [20]. The KHPS is the data with the weight of the longitudinal section of the household member, and the calculated value of weight by year is as follows.

Weighted 2011 (${\upomega}_{6\mathcal{i}}$) = Weighted 2010 (${\upomega}_{5\mathcal{i}}$) x Non-response weighted (${\phi}_{6/5\mathcal{i}}^{-1}$) x Post adjustment weighted (ω_*p*_).

Weighted 2012 (${\upomega}_{6\mathcal{i}}$) = Weighted 2011 (${\upomega}_{5\mathcal{i}}$) x Non-response weighted (${\phi}_{6/5\mathcal{i}}^{-1}$) x Post adjustment weighted (ω_*p*_).

Weighted 2013 (${\upomega}_{6\mathcal{i}}$) = Weighted 2012 (${\upomega}_{5\mathcal{i}}$) x Non-response weighted (${\phi}_{6/5\mathcal{i}}^{-1}$) x Post adjustment weighted (ω_*p*_).

The weights of the longitudinal section of the household member were the weights assigned to the household members who continued to participate in the survey. The non-response adjustment weights were finally benchmarked with the known statistics and subjected to post-adjustment. Post adjustment weights were adjusted using the statistical population estimation result.

Unmet healthcare needs were calculated using a complex weighted sample design. The difference between groups of categorical variables was analysed using the Rao-Scott Chi-square test. A logistic regression analysis was performed to analyse the association between unmet healthcare needs and aging factors. For all statistical analysis, *p* <  0.05 was considered statistically significant. Statistical analyses were performed using the PASW software (version 24.0; SPSS Inc., Chicago, IL).

## Results

### Unmet healthcare needs according to general characteristics

Of the 47,746 participants in 2011–2013, 8957 were 65 years of age or older. The participants included in the final analysis were 8666 excluding missing values (not responding to unmet healthcare needs).

**Fig. 1:**
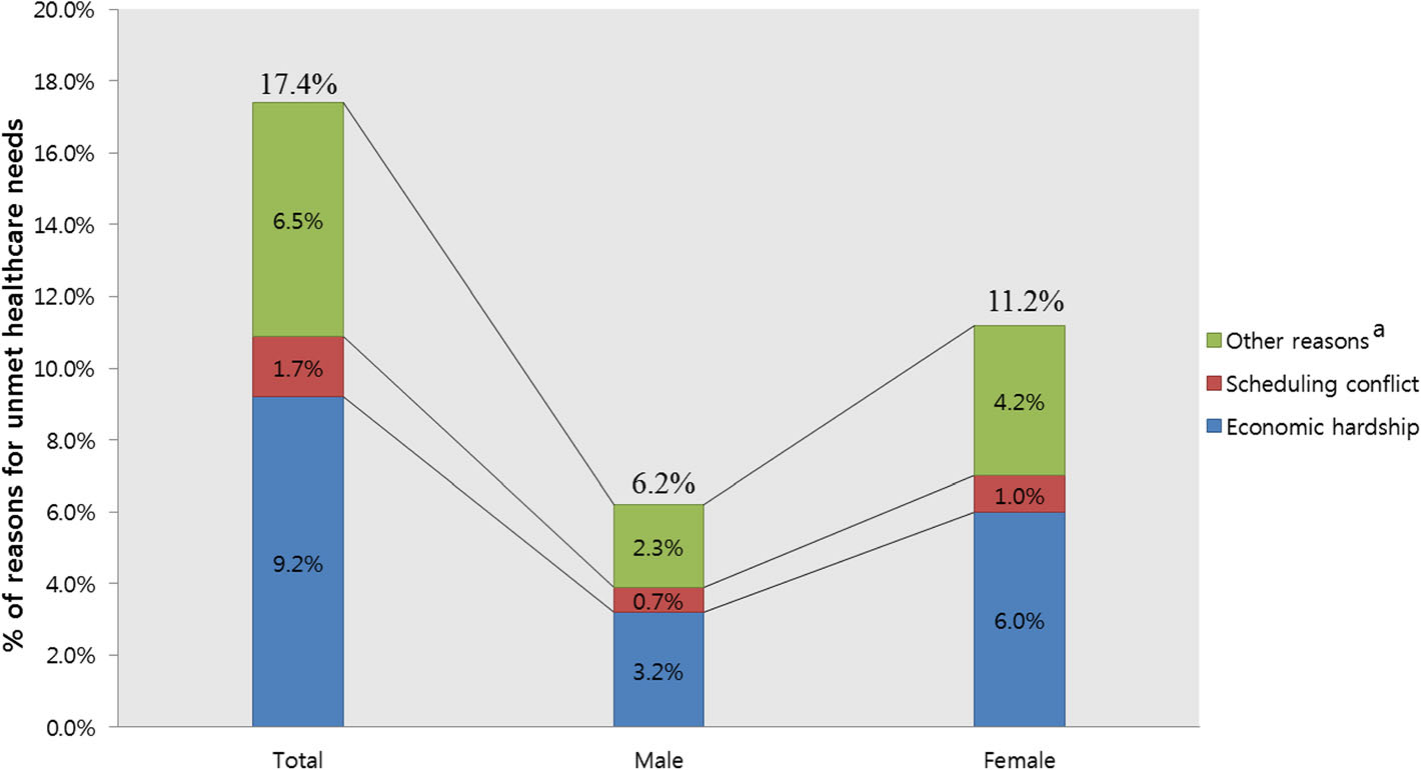
Reasons for unmet healthcare needs according to sex. ^a^inaccessible transportation, physical disabilities, problems in finding childcare, mild symptoms, lack of information about hospitals, difficulties in getting appointments at hospitals, no regular family doctor, and others

**Fig. 2:**
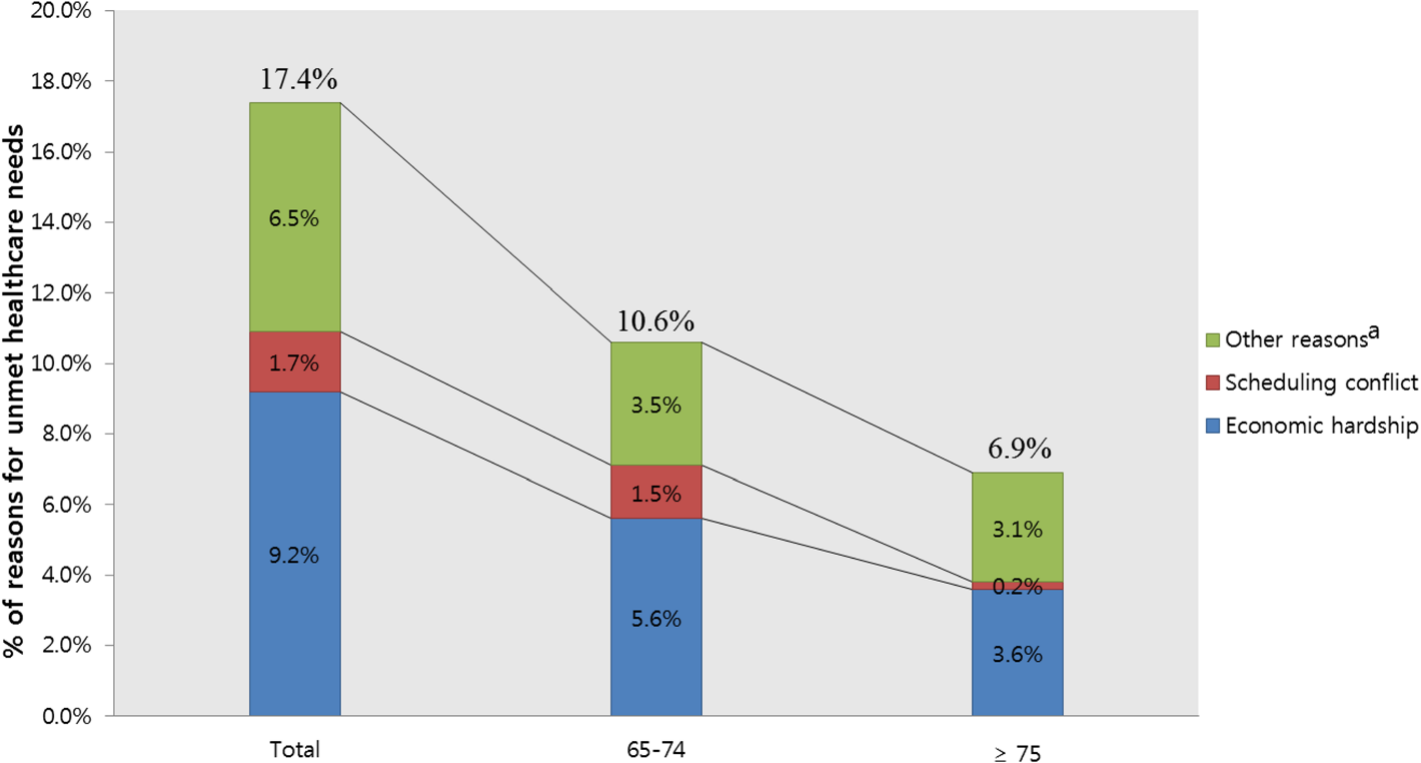
Reasons for unmet healthcare needs according to age. ^a^inaccessible transportation, physical disabilities, problems in finding childcare, mild symptoms, lack of information about hospitals, difficulties in getting appointments at hospitals, no regular family doctor, and others

Overall, 17.4% of participants (*n* = 8666) had unmet healthcare needs. Of these, 9.2% were due to economic hardship classified as accessibility, 1.7% due to scheduling conflict classified as availability of service, and 6.5% due to other reasons including acceptability of available services (Figs. 1 and 2).

Participants with met and unmet healthcare needs differed significantly in terms of sex (*p* <  0.001) and age (*p* = 0.002). Among socioeconomic characteristics, spouse (*p* <  0.001), education (*p* <  0.001), types of health insurance (*p* <  0.001), private health insurance (*p* = 0.021), and household income (*p* <  0.001) were significantly associated with unmet healthcare needs. Among health-related characteristics, self-perceived health status (*p* <  0.001), usual source of care (*p* <  0.001), and having a regular family doctor (*p* <  0.001) were significantly associated with unmet healthcare needs. Among aging characteristics, disability (*p* = 0.013), depressive symptoms (*p* <  0.001), visual impairment (*p* <  0.001), hearing impairment (*p* <  0.001), memory impairment (*p* <  0.001), and decision-making impairment (*p* <  0.001) were significantly associated with unmet healthcare needs (Table 2).

**Table 2:** Differences between general characteristics and unmet healthcare needs (n = 8666, *N* = 1.8^a^)

Characteristic	Total, % (SE)	Unmet, % (SE)	*p*-value
Participants	n = 8666, N = 1.8^a^	*n* = 1510, *N* = 0.3^a^	
Sex			
Male	41.8 (0.6)	13.9 (0.6)	< 0.001
Female	58.2 (0.6)	19.9 (0.6)	
Age			
65–74	63.3 (0.6)	16.4 (0.5)	0.002
≥ 75	36.7 (0.6)	19.1 (0.8)	
Spouse			
Yes	67.3 (0.5)	15.6 (0.5)	< 0.001
No	32.7 (0.5)	20.9 (0.8)	
Education			
Uneducated	16.3 (0.4)	24.3 (1.3)	< 0.001
Middle school or less	58.6 (0.6)	17.9 (0.6)	
High school or less	17.0 (0.4)	12.0 (0.9)	
College or more	8.1 (0.3)	10.8 (1.3)	
Types of health insurance			
National Health Insurance	91.4 (0.3)	16.4 (0.4)	< 0.001
Medicaid	8.6 (0.3)	27.3 (1.8)	
Private health insurance			
Yes	44.1 (0.6)	15.0 (0.7)	0.021
No	55.9 (0.6)	17.2 (0.6)	
Economic status			
Employed	33.5 (0.5)	16.9 (0.7)	0.394
Unemployed	66.5 (0.5)	17.6 (0.5)	
Household income			
1st quintile	39.0 (0.5)	23.3 (0.8)	< 0.001
2nd quintile	25.6 (0.5)	16.2 (0.8)	
≥ 3rd quintile	35.3 (0.5)	11.7 (0.6)	
Self-perceived health status			
Good/very good	30.0 (0.5)	10.4 (0.7)	< 0.001
Fair	39.8 (0.6)	15.8 (0.7)	
Poor/very poor	30.2 (0.5)	26.5 (0.9)	
Current smoker			
Yes	13.3 (0.4)	19.1 (1.2)	0.102
No	86.7 (0.4)	17.1 (0.5)	
Chronic diseases			
Yes	94.4 (0.3)	17.4 (0.4)	0.789
No	5.6 (0.3)	16.9 (1.9)	
Usual source of care			
Yes	52.9 (0.7)	17.3 (0.7)	< 0.001
No	47.1 (0.7)	21.2 (0.8)	
Regular family doctor			
Yes	31.8 (0.7)	15.8 (0.9)	< 0.001
No	68.2 (0.7)	20.6 (0.7)	
Disability			
Yes	15.8 (0.4)	19.8 (1.1)	0.013
No	84.2 (0.4)	16.9 (0.5)	
Depressive symptoms			
Yes	10.0 (0.3)	29.7 (1.7)	< 0.001
No	90.0 (0.3)	16.1 (0.4)	
Visual impairment			
Yes	49.5 (0.6)	22.2 (0.7)	< 0.001
No	50.5 (0.6)	12.6 (0.5)	
Hearing impairment			
Yes	29.7 (0.5)	21.8 (0.9)	< 0.001
No	70.3 (0.5)	15.5 (0.5)	
Memory impairment			
Yes	10.1 (0.3)	28.5 (1.6)	< 0.001
No	89.9 (0.3)	16.1 (0.4)	
Decision-making impairment			
Yes	4.8 (0.3)	30.4 (2.4)	< 0.001
No	95.2 (0.3)	16.7 (0.4)	

*n* unweighted sample size, *N* weighted sample size in millions

### Factors affecting unmet healthcare needs

Factors associated with unmet healthcare needs as revealed by the bivariate logistic regression analysis are presented in Table 3. The unadjusted socioeconomic and health-related factors significantly associated with unmet healthcare needs were being uneducated (OR = 1.67, 95% CI = 1.02–2.74), unemployed (OR = 1.23, 95% CI = 1.01–1.49), in the 1st quintile (OR = 2.00, 95% CI = 1.59–2.50) and in the 2nd quintile (OR = 1.40, 95% CI = 1.09–1.79), a fair (OR = 2.60, 95% CI = 2.02–3.34) and poor/very poor (OR = 1.68, 95% CI = 1.31–2.15) self-perceived health status, and without a regular family doctor (OR = 0.73, 95% CI = 0.56–0.96). After adjusting for aging, factors significantly associated with unmet healthcare needs were being over 75 years old (OR = 1.26, 95% CI = 1.02–1.57), unemployed (OR = 1.26, 95% CI = 1.04–1.54), in the 1st quintile (OR = 2.00, 95% CI = 1.59–2.51) and in the 2nd quintile (OR = 1.38, 95% CI = 1.08–1.78), a fair (OR = 2.15, 95% CI = 1.66–2.80) and poor/very poor (OR = 1.53, 95% CI = 1.19–1.97) self-perceived health status, and without a usual source of care (OR = 0.84, 95% CI = 0.66–1.08).

**Table 3:** Socioeconomic and health-related factors associated with unmet healthcare needs (n = 8666, N = 1.8^a^)

Characteristic	Odds ratio (95% confidence interval)	
	Unadjusted	Aging factor^1^ adjusted
Sex (Ref.: male)		
Female	0.91 (0.72–1.15)	0.90 (0.71–1.14)
Age (Ref.: 65–74)		
≥ 75	1.13 (0.91–1.39)	1.26 (1.02–1.57)^c^
Spouse (Ref.: yes)		
No	0.85 (0.68–1.05)	0.87 (0.69–1.08)
Education (Ref.: college or more)		
Uneducated	1.67 (1.02–2.74)^c^	1.55 (0.93–2.56)
Middle school or less	1.24 (0.80–1.94)	1.19 (0.76–1.88)
High school or less	0.99 (0.61–1.62)	1.00 (0.61–1.65)
Types of health insurance (Ref.: National Health Insurance)		
Medicaid	0.83 (0.60–1.14)	0.86 (0.62–1.19)
Private Health Insurance (Ref.: yes)		
No	0.96 (0.79–1.17)	0.96 (0.79–1.17)
Economic status (Ref.: employed)		
Unemployed	1.23 (1.01–1.49)^c^	1.26 (1.04–1.54)^c^
Household income (Ref.: ≥ 3rd quintile)		
1st quintile	2.00 (1.59–2.50)^a^	2.00 (1.59–2.51)^a^
2nd quintile	1.40 (1.09–1.79)^b^	1.38 (1.08–1.78)^c^
Self-perceived health status (Ref.: good/very good)		
Fair	2.60 (2.02–3.34)^a^	2.15 (1.66–2.80)^a^
Poor/very poor	1.68 (1.31–2.15)^a^	1.53 (1.19–1.97)^b^
Current smoker (Ref.: no)		
Yes	1.13 (0.86–1.49)	1.11 (0.87–1.47)
Chronic diseases (Ref.: no)		
Yes	0.84 (0.56–1.25)	0.76 (0.51–1.14)
Usual source of care (Ref.: yes)		
No	0.85 (0.67–1.08)	0.84 (0.66–1.08)^c^
Regular family doctor (Ref.: yes)		
No	0.73 (0.56–0.96)^c^	0.75 (0.57–0.99)

*n* unweighted sample size, *N* weighted sample size in millions

*Ref.* reference

^1^includes disability, depressive symptoms, visual impairment, hearing impairment, memory impairment, and decision-making impairment

^a^*p* < 0.001; ^b^*p* < 0.01; ^c^*p* < 0.05

The results of aging factors associated with unmet healthcare needs per unadjusted and adjusted models are shown in Table 4. In Model I (unadjusted), Model II (adjusted for sex and age), and Model III (adjusted for sex, age, and socioeconomic characteristics), the group with depression syndrome, visual impairment, hearing impairment, memory impairment, and decision-making impairment showed a higher rate of unmet healthcare needs than the group without depression syndrome, visual impairment, hearing impairment, memory impairment, and decision-making impairment.

**Table 4:** Aging factors associated with unmet healthcare needs (n = 8666, N = 1.8^a^)

Characteristic	Odds ratio (95% confidence interval)			
	Model I^1)^	Model II^2)^	Model III^3)^	Model IV^4)^
Disability (Ref.: no)				
Yes	0.99 (0.83–1.17)	1.02 (0.86–1.21)	0.99 (0.81–1.21)	0.99 (0.77–1.28)
Depressive symptoms (Ref.: no)				
Yes	1.83 (1.53–2.18)^a^	1.77 (1.48–2.12)^a^	1.63 (1.33–2.01)^a^	1.45 (1.13–1.88)^b^
Visual impairment (Ref.: no)				
Yes	1.74 (1.52–1.98)^a^	1.69 (1.48–1.93)^a^	1.74 (1.49–2.02)^a^	1.48 (1.22–1.79)^a^
Hearing impairment (Ref.: no)				
Yes	1.17 (1.02–1.34)^b^	1.19 (1.03–1.36)^c^	1.32 (1.13–1.56)^b^	1.40 (1.15–1.72)^b^
Memory impairment (Ref.: no)				
Yes	1.61 (1.31–1.97)^a^	1.57 (1.28–1.93)^a^	1.61 (1.27–2.04)^a^	1.74 (1.28–2.36)^a^
Decision-making impairment (Ref.: no)				
Yes	1.48 (1.09–2.02)^c^	1.48 (1.09–2.01)^b^	1.58 (1.10–2.25)^c^	1.04 (0.65–1.68)

*n* unweighted sample size, *N* weighted sample size in millions

*Ref.* Reference

^1)^unadjusted; ^2)^adjusted for sex and age; ^3)^adjusted for sex, age, and socioeconomic characteristics (spouse, education, insurance types, private health insurance, economic status, and household income); ^4)^adjusted for sex, age, socioeconomic characteristics (spouse, education, insurance types, private health insurance, economic status, and household income), and health-related characteristics (self-perceived health status, current smoker, chronic disease, usual source of care, and regular family doctor)

^a^*p* < 0.001; ^b^*p* < 0.01; ^c^*p* < 0.05

According to Model IV (adjusted for sex, age, socioeconomic characteristics, and health-related characteristics), the group with depression syndrome was 1.45 times more likely to have unmet healthcare needs than that without depression syndrome (95% CI = 1.13–1.88, *p* = 0.006). The group with visual impairment was 1.48 times more likely to have unmet healthcare needs than that without visual impairment (95% CI = 1.22–1.79, *p* <  0.001). The group with hearing impairment was 1.40 times more likely to have unmet healthcare needs than that without hearing impairment (95% CI = 1.15–1.72, *p* = 0.001). The group with memory impairment was 1.74 times more likely to have unmet healthcare needs than that without memory impairment (95% CI = 1.28–2.36, p <  0.001).

## Discussion

The physiological changes of the human body due to aging can reduce body composition, nervous functions, homeostatic functions, energy production, regulating functions, etc., even though an elderly person does not have a specific disease. These problems can cause gait disorders, cognitive disorders, sensory disorders, and so on [21]. These problems are also easily overlooked by natural changes related to aging, and often lead to a lack of healthcare, which may be needed for diagnosis and treatment. Unfortunately, these unmet healthcare needs also lead to further physical, mental, and social dysfunctions among older adults, thus resulting in a poorer health status and increased health inequalities [5].

This study analysed factors related to the unmet healthcare needs of older adults based on their KHPS data.

In this study, 94.4% of elderly people had chronic diseases, 15.8% had a disability, 10.0% had depressive symptoms, 49.5% had visual impairment, 29.7% had hearing impairment, 10.1% had memory impairment, and 4.8% had decision-making impairment. In France, 98.0% had at least one chronic health problem or disease, 20.0% had depressive symptoms, 10.2% had visual impairment, and 3.6% had hearing impairment [15].

Unmet healthcare needs were found in 17.4% of the participants. Of the unmet healthcare needs, we revealed that 9.2% were due to economic hardship classified as accessibility, 1.7% due to scheduling conflict classified as availability of service, and 6.5% due to other reasons including acceptability of available services. Previously, this number was reported as 0.4–13.8% for the general population [5] and 12.2–13.1% for the adult population [22, 23]. In Greece, the proportion was higher (26.3%) [2]. Consistently, an analysis of the KHPS in 2012 revealed a prevalence rate of 16.9% [24], and the elderly had the highest amount of unmet healthcare needs among all age groups.

In a Canadian study, adults reported problems of availability of services (54.9%), followed by acceptability of available services (42.8%) and accessibility (12.7%) [4].

Like past results, we revealed that economic hardship (accessibility) was a key reason for unmet healthcare needs [24, 25]; however, in past studies of young adults, ‘waiting time is too long (availability of services)’ [3] and being ‘too busy (acceptability of available services)’ [2] were deemed more vital reasons than economic hardship (accessibility). Perhaps, economic hardship is the primary concern among the elderly because of a low income due to retirement; therefore, money is spent on maintaining livelihood rather than medical expenses.

As in previous studies, the following variables were key factors associated with unmet healthcare needs: sex [2, 3, 12, 14], age [12, 15, 24, 26], spouse [2, 15], education [12, 18], types of health insurance [18], household income [12, 18, 24], self-perceived health status [3, 12, 18, 24, 26], disability [12], depressive symptoms [12, 15], and regular family doctor [2, 3]. Contrastingly, in Hwang and Choi [24], disability did not affect the unmet healthcare needs of the elderly. Other studies also found significant associations regarding private health insurance, economic status, current smoker, and chronic disease variables [15, 18, 24, 26]; while some others reported results similar to this study [3, 12, 15, 26]. These conflicting results suggest that it is necessary to establish systematic reviews or meta-analyses for these factors.

Visual and hearing impairment of the elderly has been reported to have a negative impact on depression and cognitive functioning [27] and functional state [28]. Visual and hearing impairment may affect overall physical health status as well as restraint and emotionality. In general, visual and hearing impairments are often recognized as common processes of aging and are thus easily overlooked. These problems are both mediators and direct factors in unmet healthcare needs. To minimize the health problems that may arise because of visual and hearing impairments, it is necessary to develop a policy to improve the use of medical care for periodic screening and treatment. Memory impairment and decision-making impairment can also be approached in a similar fashion since they have a negative impact on medication compliance [29] and self-care [30]. Specifically, older adults with memory and decision-making impairments are less likely to be treated than those without such impairments because of poor compliance with healthcare problems.

### Strengths and limitations

It was meaningful to investigate the relationship between health-related factors, socioeconomic factors, and unmet healthcare needs among older adults using the KHPS, which provided representative data from a large population. In addition, it was meaningful to consider socioeconomic, health-related, and aging factors. However, to get a deeper understanding of the unmet healthcare needs of elderly people, it is necessary to use diverse research methods such as focus group interviews and in-depth interviews.

## Conclusions

Healthcare delivery system in Korea has been improved in terms of medical accessibility due to the expansion of quantitative supply through civilian medical care and the supply and use of control through institution and insurance. Therefore, elderly people can now freely choose a medical institution.

In the medical insurance system in Korea, patients are free to select and use hospitals of their own choice any time. Nonetheless, the high unmet healthcare needs rate among the elderly still means that there is a barrier to access medical institutions. Socioeconomic, health-related, and aging factors negatively affect older adults’ medical use. Unlike younger adults, elderly people face additional obstacles because healthcare needs are deemed a natural part of aging. Therefore, there is a need for a senior-citizen-oriented healthcare service system that addresses these problems.

Specifically, this study proposes three aspects to improve the medical utilization rate among older adults.

First, availability of services should be improved. More than 90% of the elderly have at least one chronic disease, and there is functional decline due to aging. However, in this study, elderly people with a usual source of care and a regular family doctor were reported to have higher met healthcare needs; therefore, it is necessary to customize visiting health services for elderly people who have a disability and to establish a community-link system to maintain continuity of care after acute care.

Second, accessibility focusing on the elderly should be improved. Although the Korean government has improved medical accessibility through a changed medical delivery system, it is not easy for elderly people with functional impairment to visit medical institutions alone. Additionally, Korea is supporting those encountering catastrophic medical expenses for critical diseases and special transportation (vehicle services as ‘assisted transportation’ for those with disabilities) to provide for severe disabilities. The government supports the elderly to ride the subways and buses free of charge. However, we have no system to offer transportation for the elderly who have functional impairment without diagnosis of serious diseases. If these problems are overlooked, they can lead to complex diseases, which can lead to higher medical expenses. Therefore, to improve older adults’ medical use, it is necessary to decrease out-of-pocket expenses and expand transportation support.

Third, acceptability of available services should be improved. To improve the utilization rate of the less educated, older adults should be provided with healthcare education and targeted advertising at the medical institution and national level.

In sum, to resolve these unmet healthcare needs, it is necessary to reorganize the healthcare system in Korea to include preventive and rehabilitative services that address chronic diseases in an aged society and promote life-long health promotion.

## Abbreviations

CI: Confidence interval
KHPS: Korea Health Panel Study
OR: Odds ratio
